# Notch Signaling in T Helper Cell Subsets: Instructor or Unbiased Amplifier?

**DOI:** 10.3389/fimmu.2017.00419

**Published:** 2017-04-18

**Authors:** Irma Tindemans, Marlies J. W. Peeters, Rudi W. Hendriks

**Affiliations:** ^1^Department of Pulmonary Medicine, Erasmus MC, Rotterdam, Netherlands

**Keywords:** allergy, cytokines, delta-like ligand, Jagged, Gata3, notch signaling, Th1 immunity, Th2 immunity

## Abstract

For protection against pathogens, it is essential that naïve CD4^+^ T cells differentiate into specific effector T helper (Th) cell subsets following activation by antigen presented by dendritic cells (DCs). Next to T cell receptor and cytokine signals, membrane-bound Notch ligands have an important role in orchestrating Th cell differentiation. Several studies provided evidence that DC activation is accompanied by surface expression of Notch ligands. Intriguingly, DCs that express the delta-like or Jagged Notch ligands gain the capacity to instruct Th1 or Th2 cell polarization, respectively. However, in contrast to this model it has also been hypothesized that Notch signaling acts as a general amplifier of Th cell responses rather than an instructive director of specific T cell fates. In this alternative model, Notch enhances proliferation, cytokine production, and anti-apoptotic signals or promotes co-stimulatory signals in T cells. An instructive role for Notch ligand expressing DCs in the induction of Th cell differentiation is further challenged by evidence for the involvement of Notch signaling in differentiation of Th9, Th17, regulatory T cells, and follicular Th cells. In this review, we will discuss the two opposing models, referred to as the “instructive” and the “unbiased amplifier” model. We highlight both the function of different Notch receptors on CD4^+^ T cells and the impact of Notch ligands on antigen-presenting cells.

## Introduction

Following signals from both antigen-presenting cells (APCs) and the micro-environment, activated CD4^+^ T cells are triggered to initiate secretion of specific effector cytokines. Since the original observation in 1986 upon antigenic stimulation naive CD4^+^ T cells can differentiate into T helper 1 (Th1) or Th2 effector T cells depending on polarizing cytokine signals (1), various additional Th subsets have been recognized. These include Th9, Th17, Th22, follicular T helper cells (Tfh), and regulatory T cells (Tregs), each characterized by a unique cytokine production profile and a key transcription factor [see for recent review Ref. (2)]. These Th subsets play a crucial role in appropriate immune responses during host defense, but are also involved in the pathogenesis of inflammatory diseases (3, 4).

Th1 cells mainly produce IFN-γ and TNF-α and are associated with the elimination of intracellular pathogens. Th1 development is facilitated either by IL-12 and STAT4 or by IFN-γ, STAT1, and the key Th1 transcriptional regulator T-box-containing protein (T-bet), encoded by *Tbx21* (5). Th2 cells control helminth infections and are implicated in allergic immune responses such as allergic asthma. They are potent producers of Th2 cytokines that induce IgE synthesis (IL-4), recruit eosinophils (IL-5), and cause smooth muscle hyperreactivity and goblet cell hyperplasia (IL-13). Therefore, Th2 cells are central in the orchestration and amplification of inflammatory events in allergic asthma. The master transcription factor Gata3 is necessary and sufficient for Th2 cytokine gene expression in Th2 cells (6). Because Th2 differentiation is driven by IL-4, this raises the paradox that IL-4 is required to generate the cell type that is its major producer. But the origin of the first IL-4 required for Th2 cell induction remains unclear. While a range of cell types are able to produce IL-4, Th2 cell responses can still be generated when only T cells can make IL-4, arguing against an essential role for an external source of IL-4 (7, 8).

An accumulating number of studies suggest that the Notch signaling pathway, which also plays a crucial role in early hematopoietic development and at multiple steps of T lineage development, is essential for Th cell differentiation [for recent review see Ref. (9)]. Currently, two opposing models have been proposed that explain how Notch ligands can influence Th subset differentiation. According to the “instructive” model, Jagged and delta-like ligands (DLL) on APCs induce Th2 and Th1 differentiation, respectively (10). Alternatively, the “unbiased amplifier” model proposes that Notch ligands are not instructive but rather function to generally amplify Th cell responses (11). In this review, we will discuss these two contrasting hypotheses on the role of Notch signaling. We will focus on both Notch receptor expressing T cells and Notch ligand-expressing cells.

## The Notch Signaling Pathway

**Figure 1:**
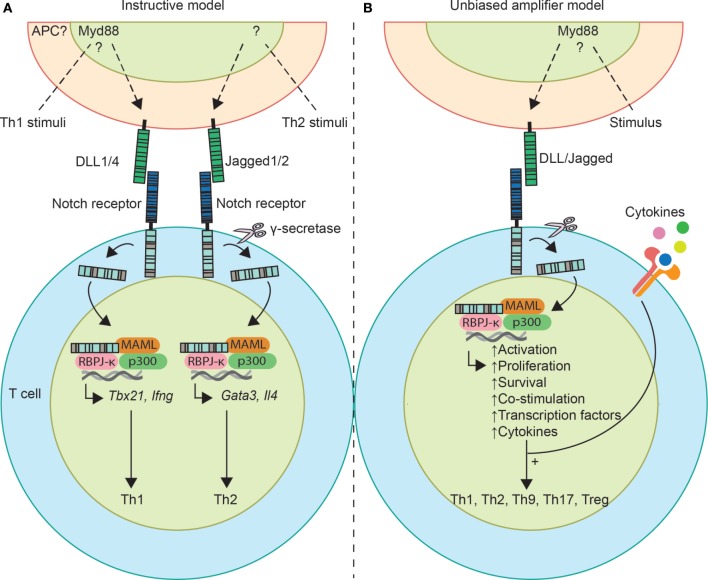
**Schematic overview of the two models describing the role of Notch signaling in T helper (Th) cell differentiation**. **(A)** According to the instructive model, Th1-stimuli and Th2-stimuli induce delta-like ligands (DLL) and Jagged ligand expression on antigen-presenting cells (APCs), respectively. Upon receptor–ligand binding, Th1 differentiation is induced by Notch intracellular domain binding and activating transcription of the Th1 transcription factor gene *Tbx21* and signature cytokine *Ifng*. For Th2 differentiation, Notch induces transcription of *Gata3* and *Il4*. **(B)** Notch ligands act as an unbiased amplifier, thereby sensitizing cells to the environment to ensure that activated CD4^+^ T cells overcome a Th cell commitment threshold. Notch induces activation, proliferation, enhances anti-apoptotic signals, and is simultaneously recruited to Th1, Th2, and Th17 genes. So, in this hypothesis Notch acts as an enabler of differentiation, whereby the outcome depends on signals of the environment, such as cytokines.

There are five Notch ligands: two Jagged (Jagged1 and Jagged2) and three DLL (DLL1, DLL3, and DLL4), which are bound by four receptors, Notch1–4. For these ligands to be functional, their ubiquitination by Mindbomb1 or Neuralized within the cell is required (12). Details of the Notch signaling pathway are discussed in various excellent reviews (13, 14). Briefly, following ligand–receptor binding, the Notch intracellular domain (NICD) is cleaved by a γ-secretase complex and translocates to the nucleus and binds to the transcription factor recombination signal binding protein for immunoglobulin Jκ region (RBPJκ; Figure 1). Finally, additional co-activating proteins are recruited, such as mastermind-like proteins (MAML1-3) and p300 to induce transcription of target genes. Notch signaling does not only induce Th lineage-defining transcription factors and cytokines (described below) but also general pathways critical for T cell activation, including IL-2 production, upregulation of the IL-2 receptor, and glucose uptake (15–18). Notch signaling potentiates phosphatidylinositol 3-kinase-dependent signaling downstream of the T cell receptor (TCR) and CD28 by inducing activation of Akt kinase and mammalian target of rapamycin, which enhances T cell effector functions and survival and allows them to respond to lower antigen doses (16, 19, 20). Notch signaling can be enhanced by the protein kinase PKCθ, which is crucial for TCR and CD28 signaling and regulation of the actin cytoskeleton (21). Moreover, upon TCR stimulation NICD interacts with other proteins in the cell in a non-canonical, RBPJκ-independent pathway that leads to NFκB activation (22, 23).

## Induction of Notch Ligands on APCs

T helper 2-promoting stimuli including helminth eggs, prostaglandin E2, cholera toxin, and allergens, such as house dust mite (HDM), birch pollen, and cockroach allergens, were shown to induce Jagged expression on APCs, as summarized in Table 1. Conversely, microbial Th1-inducing stimuli, e.g., dengue virus, respiratory syncytial virus (RSV), bacterial LPS, and the TRL9 ligand CpG, up regulate the Notch ligands DLL1/DLL4 on APCs (Table 1). Other studies, however, do not show exclusive upregulation of either DLL or Jagged molecules, but rather upregulation of Notch ligands of both families upon stimulation (10, 24–33). Interestingly, whereas surface induction of DLL requires MyD88, this is not the case for Jagged induction (10, 34–38). LPS can promote both Th1 and Th2 responses, which are MyD88 dependent and Myd88 independent, respectively, but the molecular mechanisms responsible for Jagged induction by LPS are unknown (39–41). Together, although there is also evidence that particular stimuli can induce both Th1 and Th2 differentiations, many studies support an instructive role of DLL and Jagged expression on APCs.

**Table 1:** **Evidence supporting instructive roles for Jagged and DLL in Th2 and Th1 cell differentiation, respectively**.

APC	Stimulant	Notch ligand				Additional findings	Reference
		Jag1	Jag2	DLL1	DLL4		
**Instruction of Th2 differentiation**							
GM-CSF bmDCs	PGE2 or cholera toxin	0	↑	0	0	Jag1-expressing APC induces Th2 cytokines in CD4^+^ T cells	(10)
GM-CSF bmDCs	Endotoxin^+^ OVA	↑	0	–	↑	Jag1-FC (but not siRNA-Jag1) enhances AHR, eosinophilia and Th2 cytokine production	(30, 31)
GM-CSF bmDCs	Cholera toxin	–	↑	–	0	c-kit deficient DCs lack Jag2 (but not DLL4) and induce reduced Th2 inflammation and AAI	(29)
	CpG	–	0	–	↑		
GM-CSF bmDCs	LPS + OVA/GP peptide	–	–	–	–	DCs lacking Mib1 show impaired Th2 but not Th1 differentiation *in vitro*	(28)
GM-CSF bmDCs	CpG RSV	–	–	–	↑	Blocking of DLL4 induces increased Th2 cytokine secretion and AHR	(36)
GM-CSF bmDCs	Cockroach allergen	↑	0	↑	↑↑	DLL4 suppresses Th2 cytokines and blocking of DLL4 induces increased Th2 cytokine secretion and AHR	(27)
GM-CSF bmDCs	OVA	0	0	0	↑	DCs, pretreated with DLL4, induce reduced AHR and AAI	(42)
	OVA + DLL4	↑	0	↑	↑		
GM-CSF bmDCs	Derp7	↑↑	–	–	–	Derp7 induces IL-4 secretion by CD4^+^ T cells	(43)
	LPS	↑	–	–	–		
Human BDCA1^+^ mDCs	–	–	–	–	–	Jag1 expression correlated with IL-4 expressing T cells	(44)
Human BDCA1^+^ mDCs	Diesel-exhaust particles (DEP)	↑	0	–	L	In a DC-CD4^+^ T cell co-culture anti-Jag1 decreases the IL-5/IFN-γ ratio	(45)
Human GM-CSF moDCs	APE	0	↑	↑	0	In a DC-CD4^+^ T cell co-culture, APE increases IL-5 and IL-10 secretion	(26)
	APE/PGE2 + LPS	0	↑	↓	↓		
**Instruction of Th1 differentiation**							
–	αCD28αCD3^+^ Delta1-Fc	–	–	–	–	Delta1-Fc up regulates T-bet and IFN-γ expression in T cells	(46)
GM-CSF bmDCs	LPS	↑	0	–/↑	↑	DLL1 expressing APCs induce IFN-γ producing CD4^+^ T cells *in vitro*	(10, 28)
CD11c^+^ GM-CSF bmDCs	RSV	0	0	–	↑	MyD88^−/−^ DCs have reduced DLL4 and cannot induce IFN-γ in CD4^+^ T cells	(35)
GM-CSF bmDCs	*P. acnes* CpG	–	–	–	↑	DLL4 promotes Th1 development by inhibition of IL-4 production in T cells	(38)
GM-CSF bmDCs	TMEV	–	–	–	↑	Blocking of DLL4 induces decreased Th1 cytokines in demyelinating disease	(47)
Splenic DCs (CD11c^+^CD8^−^)	LPS	L	L	L	↑	DLL4 expressing APCs induce IFN-γ (but not IL-4) in CD4^+^ T cells *in vitro*	(37)
CD11c^+^ DCs, CD19^+^ B cells	MOG35-55 peptide in CFA	↑	–	↑	–	DLL1-Fc increases Th1 cells, anti-Delta1 antibodies decrease Th1 cells; anti-Jag1 antibodies worsened EAE	(24, 48)
		0	–	↑	–		
Unknown	MOG(35–55)/CFA	–	–	–	–	DLL4-blockade decreases IFN-γ and TNF-α, promotes IL-4 production by T cells and decreases CNS inflammation	(49, 50)
Human GM-CSF moDCs	LPS	↑	L	0	↑	Expression of DLL4 correlated with IFN-γ inducing capabilities of DCs	(33)
Human BDCA1^+^ mDCs	R-848	↓	L	L	↑	Jag1 expression negatively correlated with IFN-γ expressing T cells	(44)
Human monocytes, macrophages, GM-CSF moDCs	Dengue virus	L	L	↑	0	DLL1 induces IFN-γ but not IL-4 production by CD4^+^ T cells *in vitro*	(51)
		L	L	↑	↑		
		L	L	↑	↑		
Human CD1c^+^ DCs and pDCs	R-848	–	–	–	↑	DLL4-blockade decreases IFN-γ and IL-17 expressing CD4^+^ T cells *in vitro*	(52)

*AAI, allergic airway inflammation; AHR, airway hyperreactivity; APE, aqueous birch pollen extract; bmDC, bone marrow-derived DC; CFA, complete Freund’s adjuvant; CNS, central nervous system; DLL, delta-like ligand; EAE, experimental autoimmune encephalomyelitis; GP, viral glycoprotein peptide; Jag, Jagged; mDCs, myeloid immature DCs; Mib, Mindbomb; moDCs, monocyte-derived DCs; RSV, respiratory syncytial virus; TMEV, Theiler’s murine encephalomyelitis virus; ↑, increased; ↓, decreased; 0, unaffected, L, low expression; and –, not determined*.

## The Role of Notch Ligands in Th2 and Th1 Differentiation and Function

### Th2 Cells

Notch signaling can initiate Th2 cell differentiation by direct activation of (i) a 3′ enhancer of the *Il4* gene and (ii) an upstream promoter of *Gata3* (10, 53–55). Several studies using mice expressing a dominant negative (DN) MAML transgene have demonstrated that Notch signaling is essential for Th2 cell differentiation and function (54, 56). When γ-secretase inhibitors (GSI) were used to block Notch signaling in OVA-induced asthma or food allergy models, Th2 cytokine production by T cells was inhibited while IFN-γ production was increased (57–59). Moreover, upon gene ablation of Notch1/Notch2 or RBPJκ, IL-4 production was abrogated and functional responses against parasitical pathogens were reduced (10). At the same time, IFN-γ expression was unaffected, supporting an instructive role for Notch signaling. In line with an instructive model, DLL4 was demonstrated to have a regulatory role in Th2 responses to cockroach allergen, OVA, RSV, or *Schistosoma mansoni* egg antigen (Table 1) and in an experimental autoimmune encephalomyelitis (EAE) model (49). A protective Th1 response to RSV in the lungs was converted into an allergic Th2 response by DLL4-neutralization *in vivo* (36).

However, defective Th2 responses against the intestinal helminth *Trichuris muris* in DN-MAML transgenic mice were restored when mice received anti-IFN-γ antibodies, indicating that Notch functions to optimize rather than to initiate the Th2 response (11). Moreover, decreased Th2 responses were found when DLL4 was blocked in a mouse model for RSV-mediated allergic asthma exacerbations (60). Finally, we very recently found that whereas mice with RBPJκ-deficient T cells failed to develop HDM-driven allergic airway inflammation (AAI) and airway hyperreactivity, mice with a DC-specific conditional deficiency of both Jagged1 and Jagged2 developed normal AAI following *in vivo* HDM-exposure (32). Although most studies using bmDCs would support an instructive role for Jagged in the induction of Th2 cell differentiation and function (Table 1), our studies indicate that induction of Th2 responses in HDM-driven AAI is dependent on Jagged expression on other cell types than DCs or alternatively on cooperation between Jagged and DLL on DCs.

Taken together, although several lines of evidence indicate that DCs use the Notch pathway to instruct Th cell fates, Notch may also act as an unbiased amplifier of Th cell differentiation.

### Th1 Cells

The signature Th1 genes *Ifng* and *Tbx21* were identified as direct Notch targets (11, 61). Mice in which T cells were Notch1/Notch2 double-deficient showed impaired IFN-γ secretion by Th1 cells during *in vivo Leishmania major* parasite infection, but reports employing DN-MAML transgenic or conditional RBPJκ knockout mice demonstrated that Th1 cell function was unaffected (32, 53, 54, 56, 62). Therefore, these findings suggest that signals that regulate Th1 differentiation involve RBPJκ-independent functions of Notch. Studies using GSI showed that Th1 differentiation was impaired in an *in vivo* EAE model (11, 61). By contrast, an increase in Th1 differentiation (and a concomitant decrease in Th2 cytokine production) was seen in an OVA-driven AAI model (58). The interpretation of these apparently conflicting findings remains complicated, because effects of GSI are not limited to Notch signaling and, e.g., also involve HLA-A2 expression and cadherins (63).

The capacity of DLL1/DLL4 to induce Th1 cell differentiation is supported by many *in vitro* and *in vivo* experiments, as outlined in Table 1. For example, anti-DLL4 antibodies reduced IFN-γ and TNF-α secretion by T cells *in vivo* (47, 49, 50). DLL1-blockade decreased Th1 cell numbers in an allograft model (64). Conversely, Jagged1-Fc had no effect and anti-Jagged1 antibodies worsened EAE disease (24, 48). Gene ablation of Jagged1 or Mindbomb1, which is critical for expression of functional Notch ligands, did not affect Th1 differentiation *in vitro* (28, 30).

In conclusion, although most studies would support an instructive role for DLL1/DLL4 in Th1 induction, the role of Notch signaling in Th1 cell differentiation remains incompletely understood.

### Other T Helper Cell Subsets

Given the increasing complexity of T cell subset biology, it is not unexpected that the bipotential instructional model is not sufficient to fully explain the function of Notch signaling in Th cell differentiation. For example, Notch signaling cooperates with TGF-β to induce Th9 cell differentiation and IL-9 expression *via* Jagged2 ligation (65). The *Rorc, Il17*, and *Il23r* gene promoters are direct Notch targets and, accordingly, Th17 cell differentiation is impaired when Notch signaling is blocked (66–70). Hereby, DLL1, DLL3, and DLL4 ligands were found to be essential (49, 50, 52, 60, 71), but a role for Jagged1 remains controversial (72–74). Remarkably, addition of DLL3 enhanced Th17 differentiation *in vitro* (75), although it was shown that DLL3 cannot activate Notch in adjacent cells, but inhibits signaling when expressed in the same cell as the Notch receptor (76). Differentiation and function of Tregs require Notch signaling in T cells (77–80), whereby both DLL and Jagged ligands can promote Treg expansion (81–88). Although the key Treg transcription factor Foxp3 is a direct Notch target (89), the role of Notch in Tregs seems rather complex, because targeting of DLL4 or Treg-specific components of the Notch pathway was associated with an increase of Tregs in *in vivo* autoimmune models (49, 90, 91). Moreover, hepatocytes and plasmacytoid DCs can induce IL-10 production in T cells *via* Jagged1 and DLL4, respectively (85, 92, 93). Finally, the finding that the absence of Notch receptors on T cells or DLL4 on lymph node stromal cells resulted in a deficiency of Tfh cells (94, 95), implicates Notch signaling in Tfh cell differentiation.

## “Instructive” Versus “Unbiased Amplifier” Model

As summarized in Table 1, considerable evidence supports an “instructive model” whereby pathogens direct Th1 and Th2 differentiation *via* upregulation of DLL or Jagged ligands on DCs (Figure 1). This implies that different Notch ligands induce distinct cellular responses in T cells, largely by the same signaling components. Although it has been speculated that different ligands might induce qualitatively different signals, e.g., RBPJκ-dependent or independent, or signals that differ in strength or kinetics (96), the molecular mechanisms involved are currently unknown.

It has been shown that DLL4 induces a stronger Notch signal than DLL1 or Jagged1 (86). Also, the ability of ligands to induce Notch signaling is dependent on the glycosylation status of the extracellular domain of Notch: Notch receptors carrying *N*-acetylglucosamine preferentially signal *via* delta ligands, while Jagged binding is inhibited (97). Absence or overexpression of Fringe glycosyltransferase proteins alters Th1 and Th2 differentiation (60, 98). Another possibility would be that different ligands preferentially activate different Notch receptors, which may each have unique downstream nuclear targets to induce distinct cellular programs. Indeed, it has been reported that whereas Notch1 and Notch2 activate Th2 differentiation, Notch3 promotes Th1 differentiation and IFN-γ production (46, 53). The expression of all these Notch receptors is induced on T cells upon TCR stimulation (62, 99, 100). Because different NICDs have different target gene preferences (101), distinct ligand–receptor combinations may produce quantitatively or qualitatively distinct signals (102). However, this is not supported by the findings that both Th1 and Th2 differentiation is affected in T cells that are Notch1/Notch2 double-deficient (53, 62) and that retroviral expression of Notch1 as well as Notch3 was associated with increased Th1 responses (46, 61). This issue is further complicated by the observation that individual Notch receptors are up regulated with different kinetics (103). It is, therefore, conceivable that they have distinct functions depending on the phase of the response.

Several studies are in apparent conflict with the “instructive model.” For example, DLL were reported to promote Th2 responses or Jagged ligands were implicated in Th1 induction (60, 104). Neither Jagged1 nor DLL1 could instruct Th2 or Th1 cytokine differentiation *in vitro* in the absence of polarizing cytokines (105). Importantly, Bailis et al. showed that Notch signaling simultaneously induced Th1, Th2, and Th17 gene transcription, also under polarizing conditions that were described to favor only one of the differentiation outcomes (11). In addition, Notch signaling *via* DLL4 was shown to boost antigen sensitivity of CD4^+^ T cells *via* promoting co-stimulatory signals in T cells (16). Together, this would suggest that Notch acts as a co-stimulating factor that orchestrates multiple Th cell programs by sensitizing cells to exogenous cytokines, thereby ensuring that activated CD4^+^ T cells overcome a Th cell commitment threshold. In support of a role for Notch as an unbiased amplifier (Figure 1), Notch signaling was shown to be required for optimal T cell expansion, CD25 and IL-2 induction *in vitro* of both Th1 and Th2 cells (15, 16, 18, 105). Finally, Notch signals promote survival by enhancing anti-apoptotic signals and glucose uptake (17, 106).

It is conceivable that minor differences in experimental design or conditions form the basis of the discrepant results that support one of the two opposing models for Notch function in Th differentiation. Many studies on Notch ligands on APCs have employed GM-CSF cultured bmDCs (Table 1), which were recently shown to contain not only DCs, but also monocyte-derived macrophages (107). In our own studies, we found that Jagged expression was required for the induction of a Th2 response in the lung when *in vitro* HDM-pulsed bmDCs were used for allergen sensitization, but not when mice were *in vivo* sensitized by endogenous airway DCs (32). Moreover, studies are complicated by the finding that Notch ligands are not only induced on DCs, but also on macrophages, B and T cells, or lymph node stromal cells (24, 95, 99, 108). Stimulation *via* CD46 and CD3 was shown to up regulate Jagged1 on human T cells (109), suggesting that T cells can provide Notch signals to each other. However, it is of note that normally several mechanisms, including lateral inhibition, are used to regulate Notch activity when similar cell types express both ligand and receptor. By lateral inhibition signal-sending cells actively repress their Notch signaling pathway (110), which would hamper concerted Notch-mediated differentiation and polarization of adjacent T helper cells. Finally, Notch receptors can become activated independent of ligand binding (111). Indeed, spontaneous Notch cleavage has been observed upon TCR triggering (15, 18, 22). Ligand-independent Notch signaling would also be supported by the recent identification of a PKCθ-dependent mechanism that enhances Notch activation (21). More experiments targeting Notch ligands in various cells types are required to determine how the Notch signaling pathway is activated in T cell subsets *in vivo*.

Another concern is that some gain-of-function approaches, involving overexpression of Notch receptors or ligands, may be associated with strong or prolonged, less physiological Notch signals. In this context, it is interesting that variable Notch signal strength allows induction of distinct responses by the same signaling pathway (112, 113), paralleling previous experiments demonstrating Th1 or Th2 cells are induced by strong or weak TCR signals, respectively (114, 115). Therefore, in studies on the effects of Notch ligands on Th differentiation, it may be critical to use a range of antigen doses. Finally, since it has recently been shown that Th2 inflammation also crucially involves IL-4-producing Tfh cells (116, 117), findings of impaired *in vivo* Th2 cell differentiation may point at Tfh rather than Th2 defects and should, therefore, be interpreted with care.

## Conclusions and Future Directions

Given the increasing number of characterized Th subsets, it is unlikely that Notch signaling simply acts as a bimodal molecular switch for the induction of either Th1 and Th2 differentiation, based on DLL and Jagged expression on DCs, respectively. Nevertheless, many studies described above support the notion that individual Notch ligands have differential effects on Th cell differentiation, which cannot be explained by the unbiased amplifier model. The two models, however, may not necessarily be mutually exclusive. Effects of Notch signaling could be quite different during induction and during maintenance of Th subset differentiation. Moreover, the finding that there is quite some plasticity between Th subsets (2) and that Th2 differentiation may involve a Tfh phase has further complicated the role of Notch signaling in Th differentiation. We also conclude that the elucidation of the role of Notch ligands on particular cell types requires comprehensive *in vivo* studies, using cell-specific knockout of individual Notch ligands or combinations.

Since Notch signaling is involved in the differentiation of basically all Th subsets, it could serve as a potential therapeutic target, for example, by inhibiting Th2 responses in allergies or Th1/Th17 responses in autoimmune diseases. However, because effects of GSI are not limited to Notch signaling, it will be valuable to develop more specific compounds targeting Notch signaling components. Indeed synthetic, cell-permeable stabilized peptides that target a critical protein–protein interface in the Notch transactivation complex (118–120) as well as specific antibodies that target Notch receptors (121–123) or Notch ligands (24, 124) have been designed. Promising results were obtained with Notch pathway blocking antibodies in cancer patients (125) and future studies should explore whether these antibodies are beneficial for allergic or autoimmune patients.

Interestingly, GSI administration during only the challenge in asthma models was sufficient to decrease Th2 cytokine production (58, 59). These findings imply that Notch signaling is not likely critical to initiate IL-4 production in activated T cells and thus the initial source of IL-4, for example in AAI, remains unclear. While several cells including basophils, Tfh cells, NKT cells, and ILC2 are capable of producing IL-4 (55, 116, 126–131), mice deficient for NKT cells, ILC2, or basophils are still capable of inducing Th2 responses (132–134), suggesting that IL-4 production by Tfh cells could be crucial for Th2 cell induction. Nevertheless, the finding that in animal models allergic disease symptoms are reduced by GSI administration during challenge only indicates that Notch signaling is important in maintaining rather than inducing Th2 cell responses. This makes Notch signaling an interesting target for development of therapeutic strategies in allergic asthma.

## Author Contributions

IT and MP performed the literature research, wrote the paper, and designed the figure. RH critically revised the manuscript.

## Conflict of Interest Statement

The authors declare that the research was conducted in the absence of any commercial or financial relationships that could be construed as a potential conflict of interest.
